# EHMTI-0027. The neuropathic pain in vascular dementia

**DOI:** 10.1186/1129-2377-15-S1-C59

**Published:** 2014-09-18

**Authors:** D Tertan, OB Tertan

**Affiliations:** 1 Neurology, Clinical Hospital Pelican, Oradea, Romania; 2 Student, University of Medicine and Pharmacy, Cluj Napoca, Romania

## Objectives

To demonstrate that an appropriate pain control in patients with vascular dementia (VD) depends on good pain evaluation and may express an improvement in behavior and daily activities.

## Methods

56 patients were diagnosed in the last two years with advanced vascular dementia according to clinical manifestations, vascular risk factors and neuroimaging which revealed brain atrophy and multiple focal lesions in the subcortical white matter. 21 of them suffered from neuropathic pain but were unable to reliably communicate their pain. So, we used Pain Assessment in Advanced Dementia Scale (PAINAD) whose total score ranges from 0 to 10 points, including mild pain (1-3), moderate pain (4-6) and severe pain (7-10). The lesions were 34% compressive-disc prolapse in the spine, producing sciatica or cervico-brachial nevralgia, 15%. were infiltrative as paraneopastic polineuropathy and 51% due to damage to the nerve itself by an intrinsec process-diabetic, alcoholic neuropathy, postherpetic neuralgia. The pain responded well to antiseizures and antidepressant medication.

## Results

From 21 patients treated with painkillers others than opioids, 15 revealed marked cognitive and behavior improvement concerning especially apathy, depression and incontinence of affect-involuntary laughing and crying.

## Conclusion

The patients with white matter lesions, particularly those noncommunicative, but also demented patients who report less prevalent pain, must be considered at high risk for undertreatment of pain. That's why, we have to use the screening instruments to check the existence of pain, first and then to check whether the pain is neuropathic.-Leeds Assessment for Neuropathic Symptoms and Signs (LANSS) and Pain DETECT.

No conflict of interest.

